# *Streptococcus sanguinis* induces neutrophil cell death by production of hydrogen peroxide

**DOI:** 10.1371/journal.pone.0172223

**Published:** 2017-02-21

**Authors:** Ryuichi Sumioka, Masanobu Nakata, Nobuo Okahashi, Yixuan Li, Satoshi Wada, Masaya Yamaguchi, Tomoko Sumitomo, Mikako Hayashi, Shigetada Kawabata

**Affiliations:** 1 Department of Oral and Molecular Microbiology, Osaka University Graduate School of Dentistry, Suita-Osaka, Japan; 2 Department of Restorative Dentistry and Endodontology, Osaka University Graduate School of Dentistry, Suita-Osaka, Japan; 3 Center for Frontier Oral Science, Osaka University Graduate School of Dentistry, Suita-Osaka, Japan; Hospital for Sick Children, CANADA

## Abstract

*Streptococcus* is the dominant bacterial genus in the human oral cavity and a leading cause of infective endocarditis. *Streptococcus sanguinis* belongs to the mitis group of streptococci and produces hydrogen peroxide (H_2_O_2_) by the action of SpxB, a pyruvate oxidase. In this study, we investigated the involvement of SpxB in survival of *S*. *sanguinis* in human blood and whether bacterial H_2_O_2_ exhibits cytotoxicity against human neutrophils. Results of a bactericidal test with human whole blood revealed that the *spxB* mutation in *S*. *sanguinis* is detrimental to its survival in blood. When *S*. *sanguinis* strains were exposed to isolated neutrophils, the bacterial survival rate was significantly decreased by *spxB* deletion. Furthermore, human neutrophils exposed to the *S*. *sanguinis* wild-type strain, in contrast to those exposed to an *spxB* mutant strain, underwent cell death with chromatin de-condensation and release of web-like extracellular DNA, reflecting induction of neutrophil extracellular traps (NETs). Since reactive oxygen species-mediated NET induction requires citrullination of arginine residues in histone proteins and subsequent chromatin de-condensation, we examined citrullination levels of histone in infected neutrophils. It is important to note that the citrullinated histone H3 was readily detected in neutrophils infected with the wild-type strain, as compared to infection with the *spxB* mutant strain. Moreover, decomposition of streptococcal H_2_O_2_ with catalase reduced NET induction. These results suggest that H_2_O_2_ produced by *S*. *sanguinis* provokes cell death of neutrophils and NET formation, thus potentially affecting bacterial survival in the bloodstream.

## Introduction

*Streptococcus sanguinis*, a member of the mitis group of streptococci, is an initial colonizer on tooth surfaces and promotes dental biofilm formation in an oral environment [1,2]. The mitis group of streptococci is comprised of that and other oral streptococci, such as *Streptococcus gordonii*, *Streptococcus oralis*, and the human pathogen *Streptococcus pneumoniae* [3], and is the leading cause of community-acquired infective endocarditis [4,5]. *S*. *sanguinis*, originally isolated from a patient with subacute bacterial endocarditis [6], has been shown to be related to bacteremia caused by dental treatment, daily toothbrushing, and ongoing dental lesions [7]. Hence, epidemiological data may have clinical relevance to show the possibility that *S*. *sanguinis* is endowed with the ability to extend its survival in blood.

Neutrophils, the most abundant type of phagocyte present in blood, constitute an essential part of the host innate immune system and possess various strategies to combat invading bacteria [8]. In addition to phagocytosis, neutrophils also kill bacteria in an extracellular manner by the use of neutrophil extracellular traps (NETs), which are DNA-based web-like structures possessing antimicrobial components, including histones, defensins, myeloperoxidase, and neutrophil elastase, and have functions to ensnare and kill pathogens [9]. Several lines of evidence have indicated that either *in vitro* infection with a pathogenic bacterial strain or stimulation with agents such as interleukin 8, lipopolysaccharide, and phorbol 12-myristate 13-acetate (PMA) trigger the active process, by which NET-associated cell death, i.e., NETosis, and subsequent release of NETs are induced [10,11]. Despite being dependent on a stimulus, reactive oxygen species are indispensable for NET formation, as neutrophils defective in NADPH oxidase activity are not able to undergo NETosis in response to PMA [10]. In PMA-induced NETosis, following activation of the Raf-MEK-ERK pathway, citrullination of histone occurs in a manner dependent on peptidyl arginine deiminase type 4 (PAD4) [12]. Resistance by the mitis group of streptococci to neutrophil bactericidal activity has been indicated as a potential virulent determinant in an endocarditis infection model, thus the ability of *S*. *sanguinis* to combat neutrophil killing strategies including NETs is most likely associated with development of vascular diseases, such as infective endocarditis [13]. However, the detailed mechanism by which *S*. *sanguinis* evades the immune system remains unclear.

Oral streptococci, including *S*. *sanguinis*, metabolize carbohydrates and produce lactate and pyruvate via glycolytic pathways. Under aerobic growth conditions, pyruvate is preferentially converted to acetyl-CoA and acetyl phosphate [14]. The pyruvate oxidase SpxB, encoded by a gene that is considerably conserved among the majority of oral streptococci [15], catalyses the conversion of pyruvate, inorganic phosphate, and oxygen to hydrogen peroxide, carbon dioxide, and acetyl phosphate [14], with acetyl phosphate serving as a precursor for ATP biosynthesis [16]. The mitis group of streptococci produces H_2_O_2_ at concentrations ranging from 0.7–1.6 mM in mixed-species biofilm [17], which is antagonistic against *Streptococcus mutans* present in oral biofilm [18]. This production of H_2_O_2_ is mostly attributable to SpxB enzymatic activity. *S*. *pneumoniae* SpxB has been implicated in carbohydrate metabolism, modulation of fatty acid composition in the cell membrane, resistance to oxidative stress, biofilm formation, induction of competence, and bacterial virulence [19–26]. The involvement of SpxB in cytotoxicity and virulence has been investigated using animal infection models and *in vitro* cytotoxicity assays, which showed that H_2_O_2_ produced by *S*. *pneumoniae* reacts with host nitric oxide to generate toxic peroxynitrite, which causes cellular damage, such as that seen in pneumococcal meningitis [27]. Furthermore, pneumococcal H_2_O_2_ also impedes the ciliary beat of human epithelium, which may contribute to the pathogenesis of respiratory tract infections [28]. Additionally, Rai *et al*. [29] reported that H_2_O_2_ produced by *S*. *pneumoniae* induces DNA double strand breaks in human alveolar epithelial cells, followed by induction of apoptosis. It has also been suggested that other mitis group streptococci including *S*. *sanguinis* exert virulence via H_2_O_2_ cytotoxic activity [30].

We previously reported that H_2_O_2_ produced by *S*. *oralis* and *S*. *sanguinis* was sufficient to induce cell death of epithelial cells and THP-1 macrophages [30–32]. More recently, we also demonstrated that H_2_O_2_ produced by *S*. *oralis* induces lysosomal impairment of infected RAW 264 macrophages, leading to cell death [33]. Of note, the cytotoxicity of H_2_O_2_ and its induction of cell death were found to be dependent on its concentration, and varied among cell types. In the present study, we examined whether H_2_O_2_ produced by *S*. *sanguinis* exhibits cytotoxicity against neutrophils, as well as its contribution to bacterial survival in blood and evasion from killing by neutrophils.

## Materials and methods

### Bacterial strains and culture conditions

The wild-type *S*. *sanguinis* strain SK36 (kindly provided by Dr. Kilian) [34], hereafter referred to as WT, and its derivatives were routinely cultured in Todd-Hewitt broth (TH, Becton Dickinson, NJ, USA) at 37°C. Overnight cultures were diluted 100-fold in 3 ml of TH broth and cultured at 37°C in a normal atmosphere until the optical density at 600 nm (OD_600_) reached 0.9 (late-exponential phase), unless otherwise stated. For infection of neutrophils, bacteria were washed with PBS, then the OD_600_ value of the suspension was adjusted to 0.2 using RPMI1640 medium (Wako, Japan).

### Construction of *spxB-*deletion mutant strain of SK36

Construction of *spxB*-deletion (KO) and revertant (Wr) strains of WT was performed using the temperature sensitive shuttle vector pSET6s, as previously described [35]. Briefly, the upstream and downstream flanking region DNAs of *spxB* were connected by overlap PCR, and cloned into pSET6s, then the resulting constructs were transformed into SK36 using a synthetic competence stimulating peptide. In the presence of chloramphenicol, a merodiploid mutant strain was created after the initial allelic replacement, and then resolved to possess either mutant or wild-type alleles after the second allelic replacement by decreasing the culture temperature to 28°C and removing chloramphenicol from the culture. To examine the effects of secondary mutations that may have arisen during mutagenesis, a clone possessing the wild-type allele was defined as a revertant strain and utilized in this study. Both in-frame deletion mutant and revertant strains arose from the same merodiploid ancestor. Deletion of the *spxB* gene or reversion to the wild-type allele was confirmed by site-specific PCR.

### Evaluation of H_2_O_2_ production

H_2_O_2_ production by *S*. *sanguinis* strains was evaluated following growth on agar plates, as previously reported [36]. Overnight cultures of *S*. *sanguinis* strains were diluted 100-fold in 3 ml of TH broth and then cultured until the exponential phase (OD_600_ = 0.5) at 37°C in a normal atmosphere. Next, 5-μl aliquots were spotted on brain heart infusion (Becton Dickinson) agar medium containing 1 mg/ml ferric chloride (FeCl_3_·6H_2_O, Wako) and 1 mg/ml potassium hexacyanoferrate (III) (K_3_[FeIII(CN)_6_], Wako) (henceforth, Prussian blue agar). The combination of hexacyanoferrate (III) and iron (III) ions in an aqueous solution yields blue precipitates of Prussian blue in the presence of H_2_O_2_, allowing for detection of H_2_O_2_ production around colonies by the appearance of blue halos. The plates were incubated at 37°C for 24 h, then the areas of blue halos were determined using Image J software (http://imagej.net/Welcome). The concentration of H_2_O_2_ in liquid cultures was quantified using an Amplex Red Hydrogen Peroxide/Peroxidase Assay Kit (Life Technologies, CA, USA), according to the manufacturer's protocol. Briefly, supernatants of overnight cultures were reacted with 10-acetyl-3, 7-dihydroxyphenoxazine and generation of the red oxidation product resorufin was examined by measuring absorbance at 550 nm (*A*_550_).

### Blood bactericidal test

To evaluate bactericidal activity in human whole blood, a Lancefield bactericidal assay was performed with minor modifications [37]. Human whole blood was collected from a healthy donor. Next, 180 μl of heparinized blood was mixed with 20 μl of an *S*. *sanguinis* suspension containing 1x10^5^ colony forming units (CFU). Following 0.5–3 h of incubation at 37°C on a rotary mixer, the mixture was seeded onto TH agar plates, then after 24 h of incubation, grown colonies were counted. Survival rate was calculated as follows; survival rate = recovered CFU/inoculated CFU x100.

### Neutrophil bactericidal assay

We also investigated the bactericidal activity of neutrophils, which were isolated from heparinized human blood using a previously described method [35]. Cells were suspended in RPMI medium containing 10% fetal calf serum (FCS, SAFC Biosciences, KS, USA) with or without catalase (100 units/ml, Sigma-Aldrich). Next, 5x10^5^ cells were mixed with a bacterial suspension at a multiplicity of infection (MOI) of 10, then incubated at 37°C for 1 or 3 h in an atmosphere containing 5% CO_2_. Survival rate was calculated using the same formula described above.

### Neutrophil cell death induced by *S*. *sanguinis*

Neutrophils were infected with *S*. *sanguinis* strains at an MOI of 10. As a positive control, cells were incubated with 200 nM of PMA (Wako). After incubation for 1–3 h, lactate dehydrogenase (LDH) released into culture supernatant was evaluated using a CytoTox 96 Non-Radioactive Cytotoxicity Assay kit (Promega, WI, USA), as previously described [38]. The absorbance of cell lysates treated with PBS containing 0.25% Triton X-100 (Wako) was set at 100%. Cytotoxicity was calculated using the following equation: [(absorbance of lysate of cells infected with *S*. *sanguinis*)-(absorbance of lysate of non-infected cells)]/(absorbance of cells treated with PBS containing 0.25% Triton X-100) x100 (%).

### NET formation induced by *S*. *sanguinis* and H_2_O_2_

Neutrophils (5x10^5^ cells) were seeded into each well of poly-_L_-lysine (Sigma Aldrich)-coated 8-well chamber slides (Nalge Nunc, NY, USA) and incubated for 30 min to allow the cells to attach to the well bottom. Following a medium change, cells were infected with the *S*. *sanguinis* strains at an MOI of 10. Exogenous addition of H_2_O_2_ or 200 nM of PMA served as a positive control. After 3 h of incubation, cells were fixed with PBS containing 4% paraformaldehyde at room temperature for 20 min. Next, cells were blocked overnight with PBS containing 1% bovine serum albumin (Sigma Aldrich) at 4°C and reacted with a goat anti-human elastase polyclonal antibody (1:2000, Santa Cruz Biotechnology, CA, USA) at room temperature for 1 h. After washing with PBS, cells were incubated with Alexa Fluor 594-conjugated anti-goat IgG (1:1000, Molecular Probes, OR, USA) at room temperature for 1 h. Finally, each slide was enclosed with ProLong Gold Antifade Reagent with DAPI (Life Technologies) and observed using a Carl Zeiss Axioplan 2 fluorescent microscope system.

### Detection of histone citrullination in infected neutrophils

Neutrophils (1.5x10^6^ cells) were incubated for 30 min in a 24-well polystyrene plate. Following a medium change, cells were infected with *S*. *sanguinis* at an MOI of 10. Cells treated with 200 nM PMA served as a positive control. After 1 h of incubation at 37°C, cells were suspended with Laemmli gel loading buffer [6.25 mM Tris-HCl (pH 6.8), 4% sodium dodecylsulfate, 10% glycerol, 0.005% bromophenol blue, 50 mM dithiothreitol]. Histone citrullination was detected by immunoblot analysis with a monoclonal antibody against citrullinated histone H3 (rabbit, 1:2000, Abcam, Cambridge, UK). As a loading control, β-actin was detected using an anti-β-actin antibody (rabbit, 1:2000, Cell Signaling, MA, USA). Horseradish peroxidase (HRP)-conjugated antibody against rabbit IgG (1:2000, Cell Signaling) was used as a secondary antibody. Immunoreactive bands were detected using Prime Western Blotting Detection Reagent (GE Healthcare Life Sciences, IL, USA).

### Quantitation of NETs

Neutrophils (5x10^5^ cells) were incubated for 30 min in a 96-well plate. The culture medium was changed to RPMI1640 medium with or without catalase (100 units/ml, Sigma-Aldrich). Next, cells were infected with *S*. *sanguinis* at an MOI of 10 and incubated for 1–3 h at 37°C, then washed with PBS and reacted with 50 nM of SYTOX Green (Life Technologies), a cell-impermeable DNA-staining dye, for 10 min. The fluorescence of each sample was measured at an excitation/emission wavelength of 485/535 nm, with background fluorescence for non-infected cells subtracted from each obtained value.

### Nitroblue tetrazolium test

Neutrophils were preincubated for 1 h with 20 μM Z-VAD-FMK or dimethyl sulfoxide, as a vehicle control, followed by addition of nitroblue tetrazolium (0.4 mg/ml, Sigma Aldrich) and infection with the WT strain at an MOI of 10. After a 1-h incubation at 37°C, the same volume of 0.5 M hydrochloric acid was added to terminate the reaction. Next, the plate was centrifuged at 2000 x *g* for 10min, after which the supernatants were gently discarded. Formazan was dissolved in dimethyl sulfoxide and absorbance at 550 nm was measured. Non-infected cells served as a control.

### Statistical analysis

The significance of differences between groups was evaluated using the GraphPad Prism software package (ver. 7.0). Statistical tests utilized for each experiment are shown in legends accompanying the figures. A confidence interval with a *p* value of <0.05 was deemed to indicate significance.

### Ethics statement

Human venous blood was obtained from healthy volunteers (age range, 27–31 years) with no systemic disease, and no recent history of infectious disease or medications that could alter neutrophil function. Written informed consent was obtained according to a protocol approved by the institutional review board of Osaka University Graduate School of Dentistry. The ethics committee specifically approved this study (approval no. H26-E43).

## Results

### SpxB contributes to H_2_O_2_ production of *S*. *sanguinis* SK36

**Fig 1:**
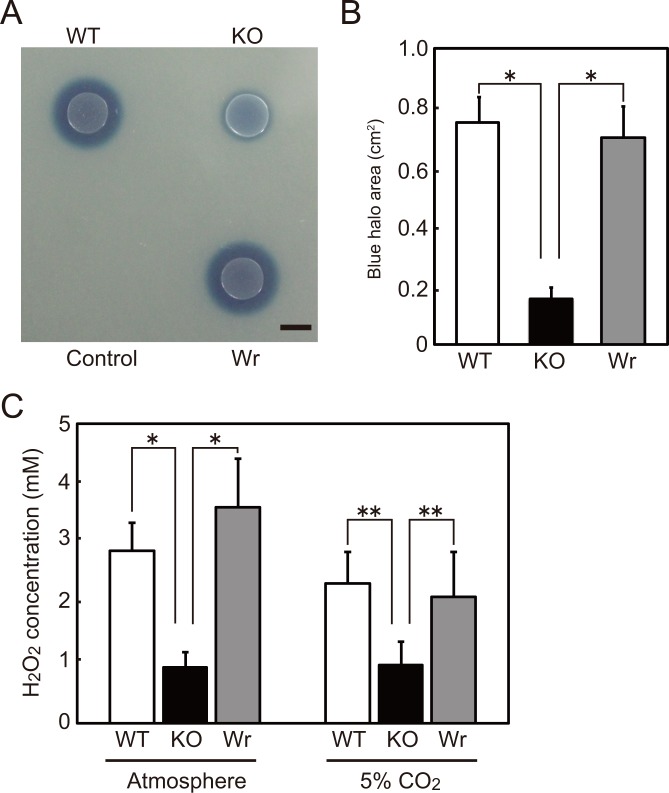
Effect of *spxB* deletion on H_2_O_2_ production by *S*. *sanguinis*. (A) H_2_O_2_ production by *S*. *sanguinis* strains was examined using Prussian blue agar plates. Overnight cultures were spotted on the plates and incubated for 24 h. BHI medium was used as a negative control. Bar, 5 mm. (B) After 24 h of incubation, the area of blue halos around colonies was determined using Image J software. Data are shown as the mean ± SD of triplicate samples from 3 independent experiments. Statistically significant differences were evaluated using one-way ANOVA and Tukey’s multiple comparison test. *p<0.01. (C) Concentrations of H_2_O_2_ in culture supernatants of *S*. *sanguinis* strains grown overnight were quantified using peroxidase reactions. Data are shown as the mean ± SD from 3 (B) or 4 independent experiments. Statistically significant differences were evaluated using two-way ANOVA and Tukey’s multiple comparison test. *p<0.01.

To investigate the role of H_2_O_2_ produced by *S*. *sanguinis* in bacterial survival in blood, we constructed *spxB* deletion mutant (KO) and revertant (Wr) strains of *S*. *sanguinis* SK36 (WT). The growth rate of the KO strain was similar to that of the WT and Wr strains (data not shown). Overnight cultures of *S*. *sanguinis* strains were spotted onto Prussian blue agar to visualize H_2_O_2_ generation. After 24 h of incubation in an atmospheric condition, the KO strain exhibited remarkably less prominent blue halos around the colonies, as compared to the WT and Wr strains (Fig 1A), and densitometric analysis indicated that the remarkable reduction in halo area was caused by *spxB* deletion (Fig 1B). H_2_O_2_ in *S*. *sanguinis* cultures was also quantified using a peroxidase reaction and Amplex Red, a colorimetric substrate, which showed that its concentration in culture supernatants of the WT and Wr strains was 2.9±0.5 and 3.6±0.8 mM, respectively (Fig 1C), thus deletion of *spxB* led to a significant decrease in H_2_O_2_ concentration (0.9±0.3 mM). Differences in H_2_O_2_ concentration between the WT/Wr and KO strains were also noted under an atmospheric condition with 5% CO_2_ (Fig 1C). Under the anaerobic condition, no halos were observed around the colonies, indicating the requirement of oxygen for SpxB activity (data not shown). Thus, in agreement with previous reports [16, 25, 39, 40], SpxB is the main enzyme responsible for oxygen-dependent H_2_O_2_ production by *S*. *sanguinis* SK36.

### H_2_O_2_ produced by *S*. *sanguinis* abrogates bactericidal activity of human whole blood

**Fig 2:**
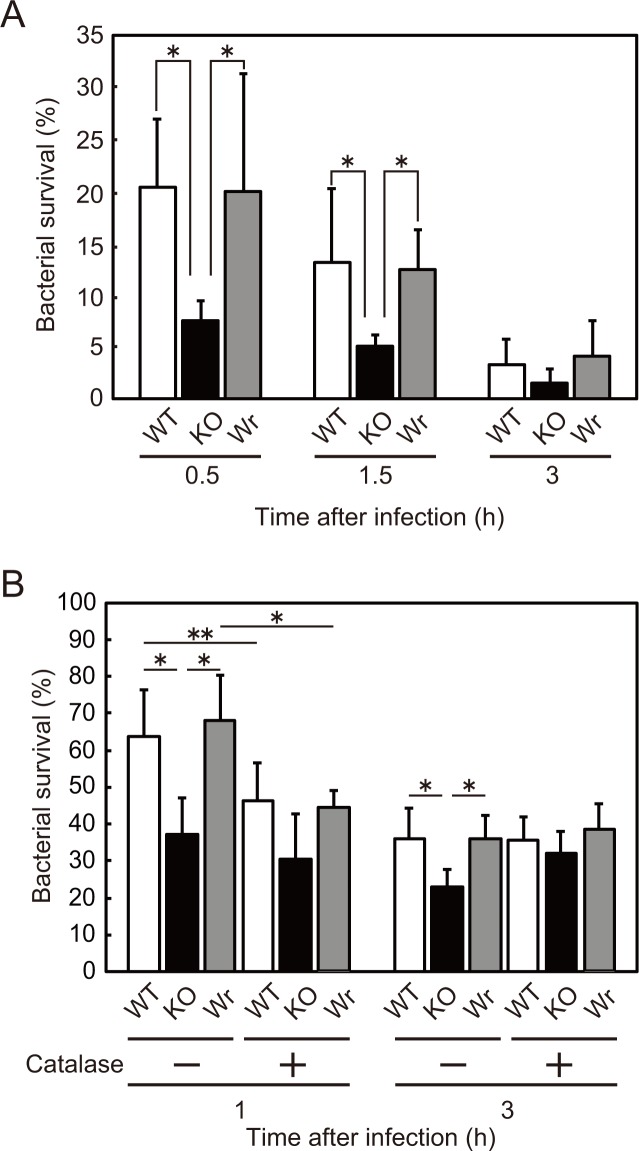
Bactericidal tests with *S*. *sanguinis* strains. (A) Human whole blood (180 μl) was mixed with the WT, KO, or Wr strain (1x10^5^ CFU). Following incubation for 0.5–3 h at 37°C, each mixture was seeded onto TH agar plates. Grown colonies were counted and the survival rate of each *S*. *sanguinis* strain is shown as % inoculum. Data are presented as the mean ± SD of triplicate samples from 3 independent experiments. Statistically significant differences were evaluated using one-way ANOVA and Tukey’s multiple comparison test. *p<0.01, **p<0.05. (B) Neutrophils were incubated with the *S*. *sanguinis* strains at an MOI of 10 with or without catalase (100 units/ml). Following incubation for 1 or 3 h at 37°C, each mixture was plated on TH agar plates. The survival rate of *S*. *sanguinis* is shown as % inoculum. Data are presented as the mean ± SD of triplicate samples from 3 independent experiments. Statistically significant differences were evaluated using two-way ANOVA and Tukey’s multiple comparison test. *p<0.01, **p<0.05.

We then examined whether SpxB is involved in survival of *S*. *sanguinis* in blood. *S*. *sanguinis* strains were exposed to heparinized human blood and the temporal survival rate was measured at 0.5, 1.5, and 3 h after infection (Fig 2A). At both 0.5 and 1.5 h after infection, the KO strain showed a significantly lower survival rate than the WT and Wr strains. At 0.5 h after infection, the survival rate of the WT and Wr strains was 20.3% and 20%, respectively, which was decreased by 3.3% and 4.0%, respectively, at 3 h after infection. On the other hand, the survival rate of the KO strain at 0.5 and 3 h after infection was 7.5% and 1.5%, respectively. These findings suggested that deletion of *spxB* renders *S*. *sanguinis* more susceptible to blood bactericidal activity.

### H_2_O_2_ produced by *S*. *sanguinis* abrogates bactericidal activity of neutrophils

Since neutrophils are the most abundant phagocytes in blood, we next examined whether their bactericidal activity is influenced by H_2_O_2_ produced by *S*. *sanguinis*. Human neutrophils with or without the H_2_O_2_-detoxifying enzyme catalase were infected with *S*. *sanguinis* strains, then the temporal survival rate of each strain was monitored for several hours. In the absence of catalase, the KO strain displayed a marked reduction in survival rate over time as compared with the WT and Wr strains (Fig 2B), findings that were fairly consistent with those obtained in the whole blood bactericidal assays. On the other hand, addition of catalase reduced the rate of survival of the WT and Wr strains at 1 h after infection, whereas there was no significant change in survival rate of the KO strain at that time point. Furthermore, the difference in survival between the WT/Wr strains and KO strain disappeared at 3 h after infection by addition of catalase. Therefore, we consider that SpxB-mediated H_2_O_2_ production and/or other phenotypic change caused by the *spxB* mutation contributes to the sensitivity of *S*. *sanguinis* to bactericidal activity of neutrophils.

**Fig 3:**
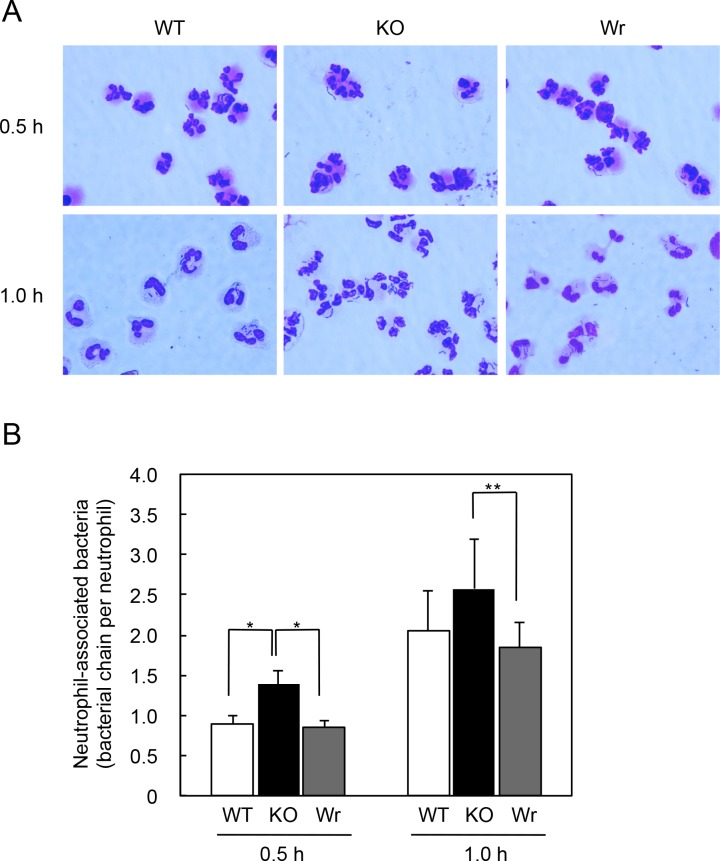
Bacterial association with human neutrophils. Human neutrophils were pre-incubated for 30 min, then infected with the WT, KO, or Wr strain at an MOI of 10. Following incubation for 0.5 or 1 h, cells were fixed with methanol and visualized by Giemsa staining. Using phase-contrast microscopy, 10 random fields (x 1000) were observed and the amount of bacteria demonstrating phagocytosis by neutrophils was determined by counting bacterial chains. (A) Representative images. (B) The numbers of bacterial chains associated with neutrophils at 0.5 and 1 h after infection are shown as the mean ± SD of 5 independent experiments. Statistically significant differences were evaluated using one-way ANOVA and Tukey’s multiple comparison test. *p<0.01, **p<0.05.

To examine whether the differences in susceptibility to bacteriocidal activity of neutrophils is caused by an effect of the *spxB* mutation on phagocytosis efficiency, the bacterial association with neutrophils was examined using infected neutrophils subjected to Giemsa staining (Fig 3). At 0.5 h after infection, the KO strain showed a greater level of phagocytosis by neutrophils as compared to the WT and Wr strains, while a difference was also observed between the KO and Wr strains at 1 h after infection. These results suggested that the survival differences between the WT/Wr and KO strains, as shown in Fig 2B, were attributable, at least in part, to different levels of phagocytosis of each strain.

### H_2_O_2_ produced by *S*. *sanguinis* is cytotoxic towards human neutrophils

**Fig 4:**
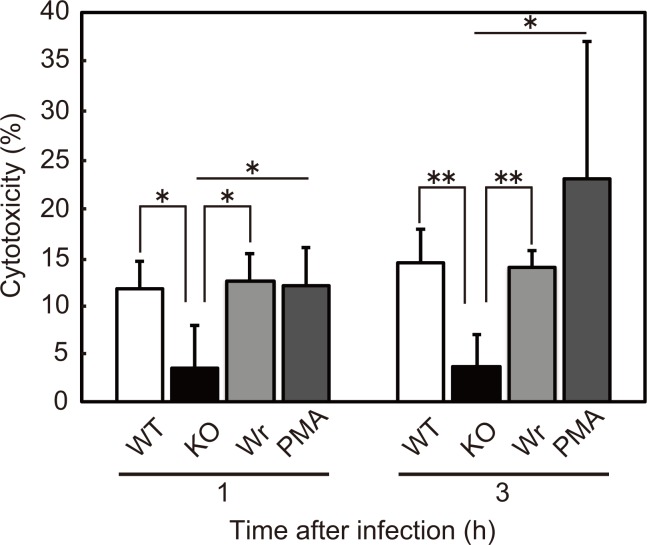
H_2_O_2_ produced by *S*. *sanguinis* showed cytotoxicity against neutrophils. Human neutrophils were mixed with the *S*. *sanguinis* WT, KO, or Wr strain at an MOI of 10. As a positive control, PMA was added at concentrations of 200 nM. Following incubation for 1 or 3 h, LDH released into culture supernatant was examined. Cytotoxicity was determined by relative absorbance, with the absorbance of cells lysed with PBS containing 0.25% Triton X-100 set at 100%. Data are presented as the mean ± SD from 3 independent experiments. Statistically significant differences were evaluated using one-way ANOVA and Tukey’s multiple comparison test. *p<0.01.

As previously reported in studies that used monocyte and epithelial cell lines, *S*. *sanguinis* induces cell death via H_2_O_2_-mediated cytotoxicity [30–33]. Thus, we examined whether the decreased bactericidal activity of neutrophils is caused by cytotoxic effects of streptococcal H_2_O_2_. Using culture supernatants of neutrophils exposed to either the *S*. *sanguinis* stains at an MOI of 10 or positive control PMA for 1 or 3 h, cytotoxicity against neutrophils was evaluated by measuring the release of LDH. That released from cells infected with WT or Wr strain was nearly the same as that from PMA-treated cells (Fig 4). On the other hand, it is important to note that the *spxB* mutation impaired the release of LDH from neutrophils, though the KO strain showed more efficient phagocytosis by neutrophils at 0.5 h after infection as compared to the WT and Wr strains, as well as at 1.0 h as compared to the Wr strain (Fig 3). These findings suggest that H_2_O_2_ produced by *S*. *sanguinis* provokes neutrophil cell death, which most likely affects the net bactericidal activity of neutrophils.

### H_2_O_2_ produced by *S*. *sanguinis* induces NET formation

**Fig 5:**
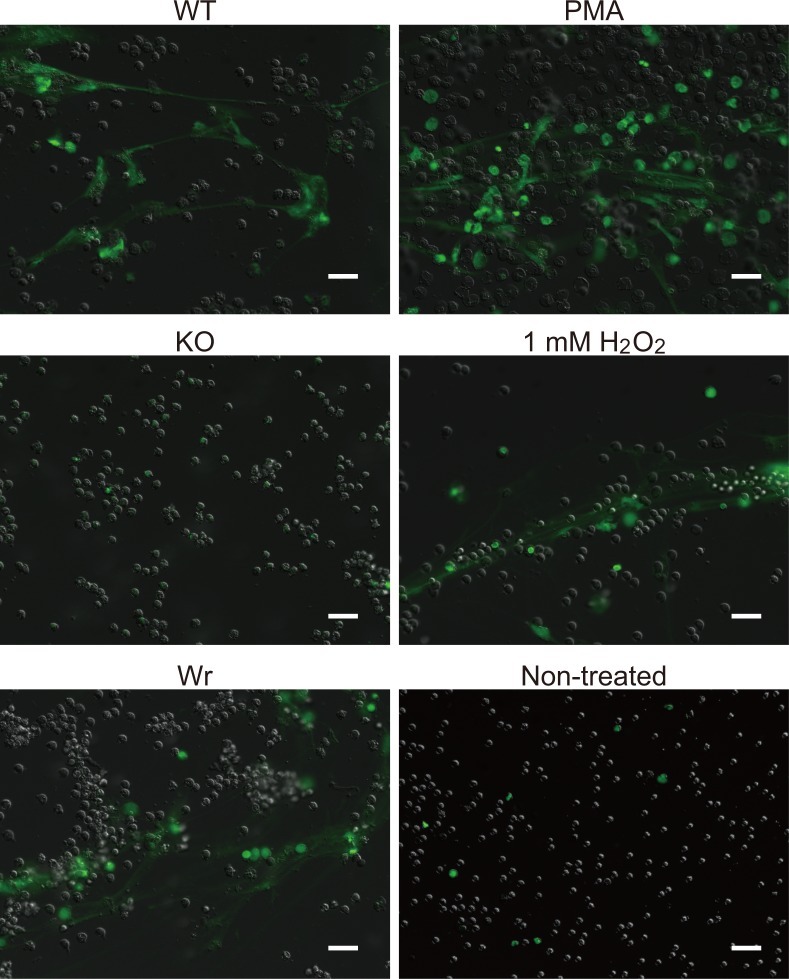
H_2_O_2_ produced by *S*. *sanguinis* induced release of NETs from infected neutrophils. Neutrophils were infected with the *S*. *sanguinis* strains at an MOI of 10. Exogenously added H_2_O_2_ (1 mM) or PMA (200 nM) served as a control. Following incubation for 3 h, cells were reacted with SYTOX Green (50 nM), then observed with a fluorescent microscope. Bar, 20 μm.

Since reactive oxygen species are involved in NET induction, we also examined whether extracellular H_2_O_2_ produced by *S*. *sanguinis* induces NETs. DNA release from neutrophils, a characteristic of NETs, was evaluated by staining extracellular DNA with SYTOX green, which is unable to penetrate an intact membrane (Fig 5). Those results revealed the presence of abundant web-like extracellular DNA from cells infected with either the WT or Wr strain, indicating NET formation, which was similar to the results obtained with PMA-treated cells. In contrast, the staining pattern was scarcely detectable in non-treated and KO strain-infected cells. Exogenously added 1 mM H_2_O_2_ also induced release of extracellular DNA. To examine the nucleus morphology of infected cells, neutrophil elastase and DNA were fluorescently labeled. Neutrophils infected with the WT or Wr strain exhibited de-condensed and non-lobulated nuclei, which were not observed in non-infected or KO strain-infected cells (S1 Fig). Cells treated with PMA or 1 mM H_2_O_2_ also showed de-condensed and non-lobulated nuclei. Therefore, it is conceivable that *S*. *sanguinis* induces NETs by cytotoxicity of self-generated H_2_O_2_.

### H_2_O_2_ produced by *S*. *sanguinis* induces citrullination of histones in infected human neutrophils

**Fig 6:**
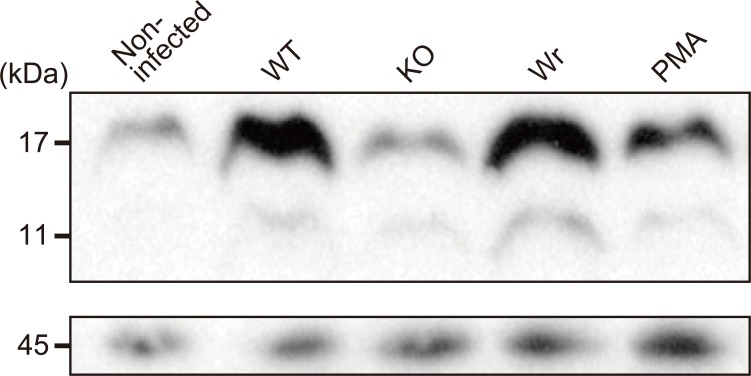
Detection of histone citrullination in *S*. *sanguinis*-infected neutrophils. Neutrophils were infected with the WT, KO, or Wr strain at an MOI of 10. Following incubation for 1 h at 37°C, cells were suspended in Laemmli gel loading buffer. Samples were immunoblotted with a rabbit anti-histone H3 antibody and HRP-conjugated anti-rabbit IgG antibody (upper panel). As a loading control, β-actin was detected using an anti-β-actin antibody and HRP-conjugated anti-rabbit IgG antibody (lower panel).

It has been reported that citrullination of arginine residues in histones H3 and H4 is a crucial step leading to loss of positive charge and subsequent chromatin unfolding during PMA-driven NET induction [32]. Immunoblot analysis revealed acceleration of the citrullination level in neutrophils infected with the WT or Wr strain, as compared with non-infected cells (Fig 6), and that level was equivalent to that induced by PMA. In contrast, neutrophils infected with the KO strain showed a background level of citrullination. Based on these results, we consider it likely that the form of neutrophil cell death induced by *S*. *sanguinis* H_2_O_2_ includes NETosis.

### H_2_O_2_ produced by *S*. *sanguinis* stimulates NET formation

**Fig 7:**
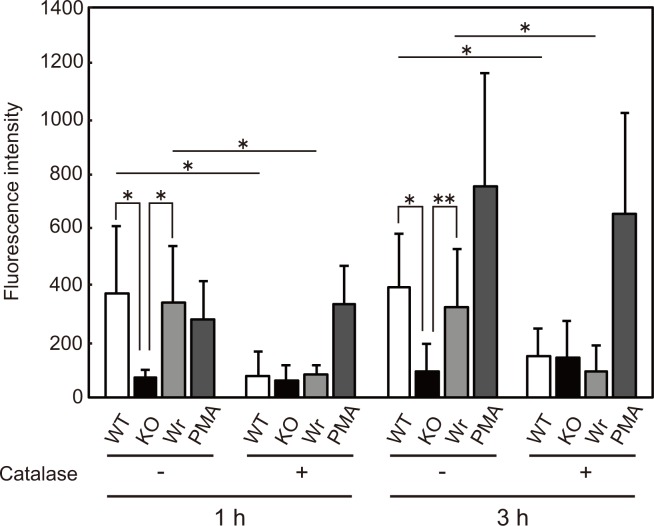
Extracellular DNA from neutrophils increased by streptococcal H_2_O_2_. Neutrophils were infected with the *S*. *sanguinis* strains at an MOI of 10 with or without catalase (100 units/ml), and cultured for 1–3 h. Neutrophils treated with 200 nM of PMA served as a control. Cells were reacted with SYTOX Green and fluorescent intensity was determined. Data are presented as the mean ± SD of triplicate samples from 3 independent experiments. Statistically significant differences were evaluated using two-way ANOVA and Tukey’s multiple comparison test. *p<0.01.

Finally, neutrophil extracellular DNA was evaluated by measuring fluorescent intensity following SYTOX green staining. The quantity of extracellular DNA from neutrophils infected with the KO strain was significantly lower as compared to that from cells infected with the WT or Wr strain (Fig 7). Furthermore, exogenous addition of catalase decreased the amount of extracellular DNA from neutrophils infected with the WT or Wr strain, whereas no effects were observed with KO strain-infected or PMA-treated cells. We also examined the amount of extracellular DNA derived from *S*. *sanguinis* cells [41]. When the *S*. *sanguinis* strains were cultured without neutrophils under the same condition used for the infection study, the level of fluorescent intensity was nearly the same as that of the background, indicating that *S*. *sanguinis* releases far lower amounts or no DNA under the present test conditions, as compared to neutrophils. These findings show that H_2_O_2_ produced by *S*. *sanguinis* mediates survival of bacteria encountering neutrophils via cytotoxicity and NET induction.

## Discussion

Routine dental treatment leads to substantial invasion of oral bacteria into the bloodstream resulting in transient bacteremia [42–44], followed by systemic dissemination and occasional onset of extra-oral diseases, including infective endocarditis and polymicrobial abscess [42, 45]. Although infective endocarditis is a rare disease with an annual incidence rate ranging from 3 to 10 episodes per 100,000 individuals in the general population, the mortality rate is considerably high at 10–26% [43, 46, 47]. It is noteworthy that *S*. *sanguinis* is one of the most common species detected in related lesions [42]. However, the molecular mechanisms involved in dissemination of *S*. *sanguinis* from the oral cavity to the bloodstream, as well as the adaptation mechanism required for persistence in both the bloodstream and vegetative lesions are largely unknown. In the light of recent findings showing that H_2_O_2_ produced by mitis group streptococci is cytotoxic towards various types of cells [30–33] and that neutrophils are related to the pathology of infective endocarditis [48, 49], we examined the cytotoxic effects of H_2_O_2_ produced by *S*. *sanguinis* against neutrophils and the role of SpxB for survival in blood in the present study.

Our findings provide important clues regarding the role of SpxB in survival of *S*. *sanguinis* organisms when encountering neutrophils. The present results showed that SpxB of *S*. *sanguinis* contributes to cytotoxicity against neutrophils (Figs 2 and 3). Based on our findings showing an inhibitory effect of catalase on cytotoxicity, we consider that H_2_O_2_ may play a role in that effect. In addition, NETosis seems to be, at least in part, the cell death form of neutrophils, as extracellular DNA and citrullinated histone were clearly detected in infected neutrophils, while they were less apparent following catalase treatment (Figs 4–7).

It was previously reported that activation of peptidyl arginine deiminase-4 is necessary for PMA-induced NET formation, which leads to replacement of charged arginine residues in histone proteins with neutrally charged citrulline residues, allowing for de-condensation of nuclear chromatin [10]. We found that induction of apoptosis was not relevant to cytotoxicity, since a broad-range apoptosis inhibitor had no effects on the ability of infected neutrophils to produce ROS, as determined with a nitroblue tetrazolium test (data not shown). Diverse cellular responses to exogenous H_2_O_2_ have been noted in mammalian cells, while distinct responses are dependent on its concentration [30–33, 50–53]. Since SpxB is involved in carbohydrate metabolism and requires oxygen for enzymatic activity, the environment for streptococcal growth undoubtedly has an impact on SpxB enzymatic activity and H_2_O_2_ production. Therefore, in contrast to the situation in blood, the level of cytotoxicity against neutrophils and possible cell death form may differ in the oral cavity, the inherent niche.

At onset, infective endocarditis becomes established on vulnerable sites of heart valves where endothelium has become exfoliated, then blood coagulum consisting of extracellular matrix proteins, plasma proteins, and platelets is subsequently formed upon contact between sub-endothelium and blood. Thereafter, disease progression is considered to be a multistep process [5], with the first step involving attachment and colonization of circulating bacteria on damaged heart tissues. Several surface proteins of *S*. *sanguinis* have been implicated in adherence to host tissues and cells [54–58]. The second step is comprised of recruitment and activation of blood monocytes, which induce production of cytokines and procoagulant factors contributing to inflammation and vegetation growth. During this process, colonized bacteria must elude the host defense system and persist in vegetation. Finally, successive accumulation of clots, leukocytes, and bacteria accelerates formation of infected vegetation. Thus, interactions among host tissues, immune cells, and bacteria underlie the pathological condition of infective endocarditis. In addition, a growing body of evidence suggests that neutrophils are also recruited to septic vegetation and NET formation is subsequently induced, thus NET is likely involved in disease pathogenesis as a scaffold for vegetation growth [48, 49, 59, 60]. It has also been reported that activated platelets promote NET induction and subsequent formation of septic thrombi in blood vessels [61]. Moreover, neutrophils play a pivotal role in defense against circulating mitis group streptococci in blood, as demonstrated by the fact that a major risk factor for bacteremia due to mitis group streptococci is severe neutropenia [62–64]. Therefore, the ability of *S*. *sanguinis* to induce NETs may be related to development of infective endocarditis.

NETs were originally reported to possess an activity that inhibits growth of ensnared bacteria. We previously showed that *S*. *sanguinis* expresses a unique cell wall-associated nuclease, designated SWAN, which allows *S*. *sanguinis* to escape from NET bactericidal activity [35]. Thus, *S*. *sanguinis* can evade bactericidal activity of neutrophils in two different ways, i.e.; direct killing of neutrophils via H_2_O_2_ cytotoxicity and degradation of NETs by SWAN nuclease activity.

Previously, exogenously added H_2_O_2_ was shown to promote neutrophil phagocytosis [65]. However, our observations of infected neutrophils indicated that the *spxB* mutant, which produced less H_2_O_2_, was more efficiently phagocytosed after 0.5 and 1 h as compared with the WT/Wr and Wr strains. Therefore, this mutant would be more susceptible to killing by phagocytosis during an early stage of infection, which is probably caused by phenotypic alteration, as reported for the *spxB* mutant of *S*. *pneumoniae* [19–26]. In agreement with those findings, in the present neutrophil bacteriocidal assay, the *spxB* mutant exhibited a lower survival rate than the WT and revertant strains. However, the difference in regard to bacterial survival rate disappeared with addition of catalase, indicating that H_2_O_2_ produced by *S*. *sanguinis* mainly influences survival rather than other potential phenotypic changes. In contrast, the cytotoxicity of *S*. *sanguinis* against neutrophils was decreased by *spxB* deletion, suggesting that the cytotoxicity against neutrophils is not mainly attributable to induction of phagocytosis-induced cell death, but rather to NET-induction mediated by H_2_O_2_.

The interaction of *S*. *sanguinis* with neutrophils was considered to be affected by phenotypic alteration modulated by SpxB. Based on studies of *S*. *pneumoniae* SpxB, in addition to reduced H_2_O_2_ production, a mutation in the *spxB* gene imparts multiple phenotypes of *S*. *pneumoniae*, which have effects on the bacterial association with neutrophils and susceptibility to their bactericidal activity, including changes in sugar utilization pattern, up-regulation of capsule production, alterations in adhesive properties such as the adhesin expression/membrane fatty acid composition, and susceptibility to H_2_O_2_ [19–26]. Although it is unclear whether these phenotypes of the *spxB* mutant are inherent in *S*. *pneumoniae* or common in *S*. *sanguinis*, we are unable to exclude the possibility that they influence, either synergistically or separately, the survival of *S*. *sanguinis*. Moreover, acetyl phosphate produced by SpxB may serve as a phosphate donor, likely with effects on the global transcriptional profile. These potential phenotypic changes induced by the *spxB* mutation in *S*. *sanguinis* remain to be defined in a future study, which is important for clarifying the impact of SpxB on *S*. *sanguinis* virulence.

The interaction of *S*. *gordonii*, a member of the mitis group, with neutrophils has been investigated. Using an experimental endocarditis model, Young Lee *et al*. reported that resistance to neutrophil killing is a potential virulence determinant of *S*. *gordonii*, since virulent strains of *S*. *gordonii* have an ability to resist the bactericidal activity of neutrophils [13]. They also found that survival of virulent strains was attributable to resistance against the intracellular killing mechanism following attachment to neutrophils and phagocytosis. In a future study, it would be intriguing to examine whether intracellular killing of *S*. *sanguinis* is affected by the *spxB* mutation.

The present results showed that the *spxB* mutation in *S*. *sanguinis* is detrimental to bacterial survival in human blood *ex vivo*. Thus far, several lines of evidence have provided insight into the role of SpxB in regard to adaptation of mitis group streptococci to various environments, including the bloodstream [20, 24, 26]. However, based on studies of pneumococcal SpxB, it remains controversial whether the *spxB* mutation is beneficial for survival in blood, as results have varied depending on the infection model and pneumococcal strain used. Syk *et al*. reported that infection with serotype 1 strains of 2 clonal complexes in both humans and mice gave rise to diverse mutations of the *spxB* gene, resulting in considerably increased virulence in mouse intravenous infection models and decreased early clearance of bacteria from the bloodstream, though nasopharyngeal colonization after intranasal infection was impaired, indicating that SpxB is required for colonization by serotype 1 strains, but negatively affects survival in blood [26]. Moreover, *spxB* expression was shown to be decreased in the lungs and bloodstream of mice infected with *S*. *pneumoniae* [66, 67]. In contrast, both invasiveness and colonization of the serotype 2 strain D39 were found to be attenuated by *spxB* deletion [20]. Therefore, adaptation of *S*. *pneumoniae* in the bloodstream likely varies among serotypes and strains. Although, typing of *S*. *sanguinis* strains is not currently feasible, antigenic variations in surface carbohydrates or proteins are obvious, as clinical isolates occasionally exhibit the Lancefield serotype H antigen [68]. Therefore, it is possible that the requirement of SpxB for survival in blood varies among *S*. *sanguinis* strains and future *in vivo* studies are needed to examine this issue. Furthermore, contradictory reports regarding the involvement of SpxB in pneumococcal survival in blood have been presented, thus the *in vivo* relevance of the current results is uncertain, since oxygen availability is low and catalase is continuously produced in blood. Nevertheless, our findings indicate that H_2_O_2_ produced by *S*. *sanguinis* has effects on the course of blood infection and modulates bacterial persistence within the host.

In summary, the present findings demonstrated that *S*. *sanguinis* evades neutrophil killing *in vitro* and counteracts innate immunity in collected blood by the action of SpxB. In a future study, together with phenotypic characterization of the *spxB* mutant, analysis using an *in vivo* infection model will be necessary to clarify the role of SpxB/H_2_O_2_ during the course of vascular disease progression. Elucidation of the interaction between *S*. *sanguinis* and host tissues, especially blood cells and endothelium, may lead to development of prophylaxis and therapeutic intervention for pathologies caused by oral streptococci.

## Supporting information

S1 Fig Neutrophil cell death induced by *S*. *sanguinis*. Neutrophils were infected with the tested *S*. *sanguinis* strains at an MOI of 10. Exogenous addition of 1 mM of H_2_O_2_ or 200 nM of PMA to neutrophils served as a control. Following incubation for 3 h and subsequent fixation, neutrophil elastase was labeled with a goat anti-human elastase polyclonal antibody and Alexa Fluor 594-conjugated anti-goat IgG. Nuclei were stained with DAPI. Bar, 20 μm.
(TIF)
