# Supplementary figures and images for: Streptococcus sanguinis induces neutrophil cell death by production of hydrogen peroxide
Source: PLoS One. 2017 Feb 21;12(2):e0172223. doi: 10.1371/journal.pone.0172223 (PMC5319702; doi:10.1371/journal.pone.0172223)

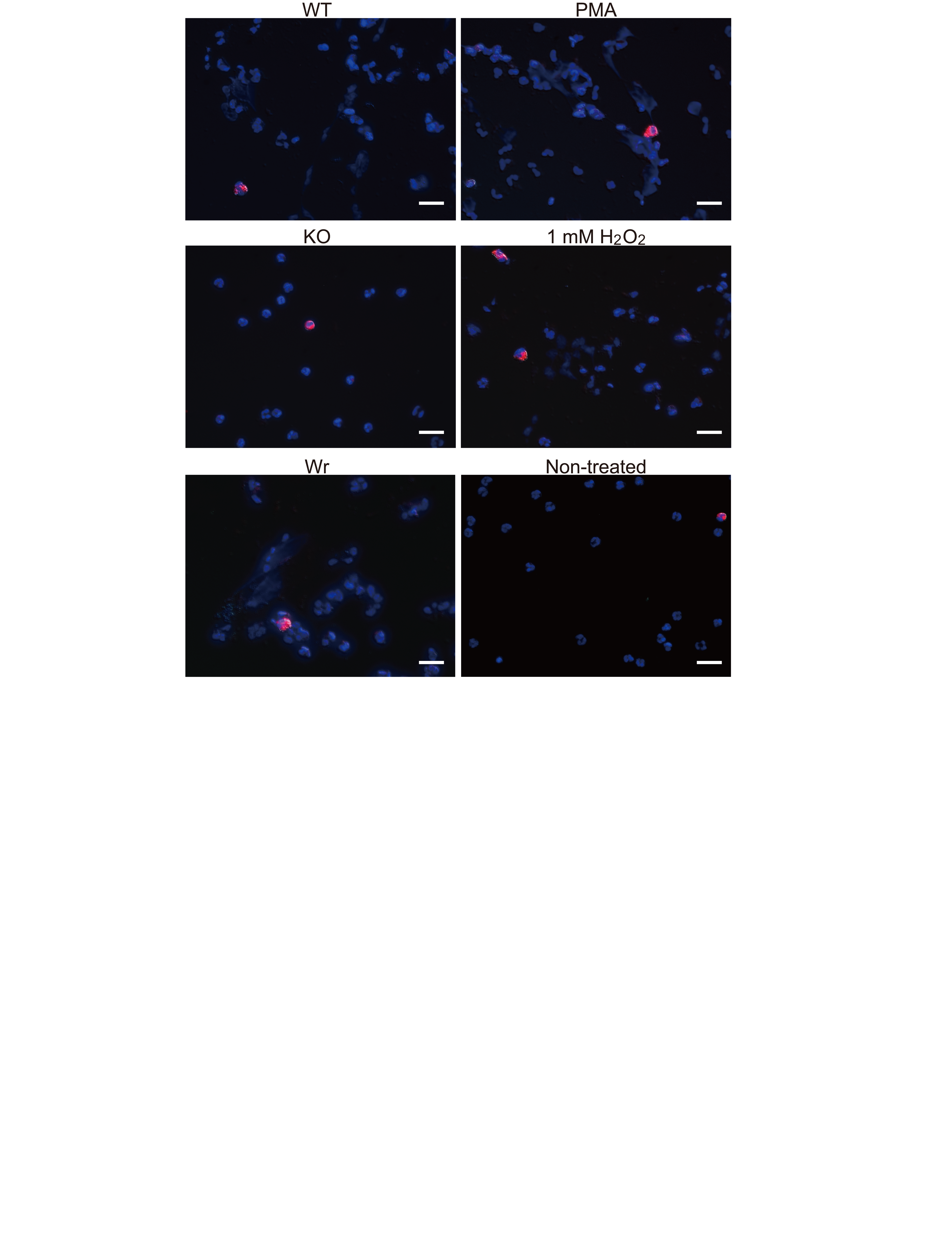

Supplement: S1 Fig — Neutrophils were infected with the tested S. sanguinis strains at an MOI of 10. Exogenous addition of 1 mM of H2O2 or 200 nM of PMA to neutrophils served as a control. Following incubation for 3 h and subsequent fixation, neutrophil elastase was labeled with a goat anti-human elastase polyclonal antibody and Alexa Fluor 594-conjugated anti-goat IgG. Nuclei were stained with DAPI. Bar, 20 μm. (TIF) [file pone.0172223.s001.tif]
